# Environmental Tuning of Homologs of the Orange Carotenoid Protein-Encoding Gene in the Cyanobacterium *Fremyella diplosiphon*

**DOI:** 10.3389/fmicb.2021.819604

**Published:** 2021-12-24

**Authors:** D. Isabel Petrescu, Preston L. Dilbeck, Beronda L. Montgomery

**Affiliations:** ^1^MSU-DOE Plant Research Laboratory, Michigan State University, East Lansing, MI, United States; ^2^Department of Microbiology and Molecular Genetics, Michigan State University, East Lansing, MI, United States; ^3^Department of Biochemistry and Molecular Biology, Michigan State University, East Lansing, MI, United States

**Keywords:** cyanobacteria, non-photochemical quenching (NPQ), orange carotenoid protein (OCP), photosynthesis, stress

## Abstract

The orange carotenoid protein (OCP) family of proteins are light-activated proteins that function in dissipating excess energy absorbed by accessory light-harvesting complexes, i.e., phycobilisomes (PBSs), in cyanobacteria. Some cyanobacteria contain multiple homologs of the OCP-encoding gene (*ocp*). *Fremyella diplosiphon*, a cyanobacterium studied for light-dependent regulation of PBSs during complementary chromatic acclimation (CCA), contains several OCP homologs – two full-length OCPs, three Helical Carotenoid Proteins (HCPs) with homology to the N-terminus of OCP, and one C-terminal domain-like carotenoid protein (CCP) with homology to the C-terminus of OCP. We examined whether these homologs are distinctly regulated in response to different environmental factors, which could indicate distinct functions. We observed distinct patterns of expression for some OCP, HCP, and CCP encoding genes, and have evidence that light-dependent aspects of *ocp* homolog expression are regulated by photoreceptor RcaE which controls CCA. RcaE-dependent transcriptional regulator RcaC is also involved in the photoregulation of some *hcp* genes. Apart from light, additional environmental factors associated with cellular redox regulation impact the mRNA levels of *ocp* homologs, including salt, cold, and disruption of electron transport. Analyses of conserved sequences in the promoters of *ocp* homologs were conducted to gain additional insight into regulation of these genes. Several conserved regulatory elements were found across multiple *ocp* homolog promoters that potentially control differential transcriptional regulation in response to a range of environmental cues. The impact of distinct environmental cues on differential accumulation of *ocp* homolog transcripts indicates potential functional diversification of this gene family in cyanobacteria. These genes likely enable dynamic cellular protection in response to diverse environmental stress conditions in *F*. *diplosiphon*.

## Introduction

Oxygenic photosynthesis, a process comprised of a series of light-driven redox reactions that results in the production of ATP and NADPH and ultimately carbohydrates, is a characteristic feature of the metabolism of cyanobacteria. Under some conditions, such as excessive light exposure or during nutrient deprivation, cyanobacteria can become unable to effectively utilize the light energy absorbed for photosynthesis. To modulate the potential negative impacts of overexcitation, while maintaining reasonable levels of photosynthesis, many photosynthetic organisms exhibit cellular responses that allow them to tune the pigment content and size of their light-harvesting complexes in response to the external photoenvironment. *Fremyella diplosiphon* (also known as *Tolypothrix* sp. PCC 7601; Yerrapragada et al., 2015) is a cyanobacterium that exhibits a form of light-dependent regulation, or photomorphogenesis, known as chromatic acclimation (Montgomery, 2016, 2017; Sanfilippo et al., 2019). *Fremyella diplosiphon* exhibits a particular form of chromatic acclimation known as complementary chromatic acclimation (CCA) that involves tuning cellular pigmentation and morphology to changes in available green versus red light (Bennett and Bogorad, 1973; Bordowitz and Montgomery, 2008). Complementary chromatic acclimation is regulated by the red/green-responsive photoreceptor RcaE (Kehoe and Grossman, 1996; Bordowitz and Montgomery, 2008).

Complementary chromatic acclimation is critical to maintain light-harvesting complexes tuned to available light quality and quantity. Despite this, cells in dynamic environments can encounter an imbalance between absorbed and utilized energy that can lead to an overly reduced photosynthetic electron transport pathway and the production of harmful reactive oxygen species (ROS; Busch and Montgomery, 2015a). The intracellular accumulation of ROS can result in cellular damage, including lipid oxidation or protein damage. This damage can ultimately result in reduced cellular fitness. To combat such potentially harmful outcomes, photosynthetic organisms possess mechanisms to protect cells from overexcitation by light. These photoprotective mechanisms include non-photochemical quenching (NPQ), or dissipation of excess absorbed light energy as heat (Montgomery, 2014).

Cyanobacteria, including *F*. *diplosiphon*, specifically use NPQ of the phycobilisome (PBS), the light-harvesting cyanobacterial antenna, under high blue-green light to protect against excess light absorption, which would cause cellular damage from the generation of ROS. NPQ is mediated in cyanobacteria by the orange carotenoid protein (OCP), which is found encoded in the genomes of numerous cyanobacteria (Melnicki et al., 2016; Bao et al., 2017a; Kerfeld et al., 2017). The OCP protein serves to mitigate absorption of excess light and to avoid the generation of ROS and associated cellular damage, through both NPQ and direct singlet oxygen (^1^O_2_) scavenging (Wilson et al., 2006, 2008; Kirilovsky and Kerfeld, 2013; Sedoud et al., 2014). Orange carotenoid protein, the isolated carotenoid-binding α-helical domain of OCP known as the red carotenoid protein (RCP; Kerfeld et al., 2003) and some Helical Carotenoid Proteins (HCPs) with homology to the N-terminal domain of OCP can function as ^1^O_2_ quenchers (Lopez-Igual et al., 2016). Recent analysis of a collection of cyanobacterial genomes has revealed that many cyanobacteria possess multiple homologs of *ocp*, including full-length variants or proteins homologous to either the N-terminal domain or C-terminal domain of OCP, and that very few cyanobacteria possess the same set of OCP homologs (Melnicki et al., 2016). Characterization of four *ocp* N-terminal domain homologs (also called helical carotenoid proteins or HCPs) from the cyanobacterium *Anabaena* sp. PCC 7120 revealed functional diversity between these proteins. One HCP protein was able to quench phycobilisome fluorescence in a manner similar to the full length OCP, whereas two were good scavengers of singlet oxygen and the fourth showed neither activity (Lopez-Igual et al., 2016). Six *ocp* homologs are found in *F*. *diplosiphon*, which include two full-length *ocp* homologs, three *hcp* homologs and one C-terminal domain-like carotenoid protein-encoding (*ccp*) gene (Bao et al., 2017b; Kerfeld et al., 2017). The physiological roles of the six *F*. *diplosiphon ocp* homologs are still under investigation. However, biochemical analyses have demonstrated distinct temperature-dependent properties and quenching of PBS fluorescence for full length OCP1 and OCP2 from *F*. *diplosiphon* (Bao et al., 2017b), despite comparable spectroscopic properties and photoactivation mechanisms (Kuznetsova et al., 2020). Additionally, initial biochemical analyses of carotenoproteins HCP2, HCP3, and CCP2 described distinct carotenoid-binding properties and excited-state dynamics (Dominguez-Martin et al., 2019, 2020; Khan et al., 2020).

Under environmental stress conditions such as cold or salt stress, photosynthetic cells often exhibit increased propensity for damage from excess light exposure. Under such conditions, cells are expected to have an increased need to protect themselves from overexcitation of PBSs. One response to such stress is for organisms to reduce cellular photosynthetic pigment content. OCP homologs are hypothesized to play critical roles in protecting cells under salt stress, cold, or other stresses.

We hypothesized that the six distinct carotenoproteins encoded by *ocp* homologs in *F*. *diplosiphon* have unique functions. Prior analyses reported transcriptional regulation of some *ocp* homolog genes, as well as distinct modes of regulation based on gene expression data showing that *OCP2* is regulated at the level of mRNA accumulation, whereas *OCP1* primarily appears to be regulated post-transcriptionally or alternatively by post-translational control by fluorescence recovery protein (FRP) or protein oligomerization (Bao et al., 2017a). The expression of *ocp1*, which is differentially regulated by sinusoidal versus fluctuating light, is also associated with differential organismal fitness under dynamic conditions (Agostoni et al., 2016). For this study, we used quantitative real-time PCR (qPCR) to measure the expression levels of the six *ocp* homologs and the *frp* gene in *F*. *diplosiphon* cultures grown under various conditions and in various mutant strains to investigate their environmental and genetic regulation. The results of this study show that patterns of expression for the *ocp* homologs and the *frp* gene fall into three classes. The first class being *ocp1* and *frp*, the second class being *ocp2* and the third class being *hcp1*, *hcp2*, *hcp3*, and *ccp2*. The distinct expression patterns suggest specialized roles for these homologs in response to different environmental conditions associated with photoacclimation of cells to dynamic light conditions to promote photoprotection, thereby supporting increased photosynthetic efficiency and organismal productivity.

## Materials and Methods

### Strains and Growth

A short-filament strain of *F*. *diplosiphon* UTEX 481, known as SF33, was used as wild-type (WT; Cobley et al., 1993); additionally *F*. *diplosiphon* strains deficient in *rcaE* (Kehoe and Grossman, 1996) and *rcaC* (Bordowitz and Montgomery, 2008) were used in this study. Cultures were maintained in BG-11 medium (Allen, 1968) with 20 mM HEPES at pH 8.0 (hereafter, BG-11/HEPES) under white fluorescent light (WL; F20T12-P/AQ) at 15 μmol m^–2^ s^–1^ at 28°C while being shaken at 175 rpm. For experiments involving red or green light, cultures were grown under red light (RL; λmax at 660 nm, LED wholesalers, model 2506RD, Hayward, CA) or green light (GL; λmax at 530 nm; Geneva Scientific LLC, Williams Bay, WI) LEDs at 10, 20, 50, or 100 μmol m^–2^ s^–1^ as indicated. Light intensities were measured with a LI-250A light meter (LI-COR, Lincoln, NE) equipped with a quantum sensor (model LI-190SA).

Cultures grown for experiments were diluted to an initial optical density at 750 nm (OD_750_) of ∼0.1 in a volume of 50 ml of BG-11/HEPES or other variants of BG-11/HEPES medium as indicated and then grown for the indicated time period (e.g., 24 h up to ∼1 week) under the indicated light conditions. Generally 18–24 h before harvesting cells for RNA extraction, the OD_750_ of each culture was checked and adjusted to ∼0.6. To measure the rapid response to stress treatments and avoid long-term growth impacts due to stress, stress treatments were applied for up to 24 h. For experiments involving cold stress, cultures were transferred to a growth chamber with a temperature of 15 or 8°C for the final 18–24 h of growth. Other samples were treated with either 10 μM of 3-(3,4-dichlorophenyl)-1,1-dimethylurea (DCMU) (v/v) or 0.03 μM of methyl viologen (MV) (v/v) for the final 24 h before harvesting cells. For salt stress or nitrogen-limitation, samples growing in BG-11/HEPES medium were harvested once the concentration reached 0.6 OD and resuspended in media with 0.2 M salt (NaCl) or lacking nitrogen then incubated for an additional 24 h. The control samples were resuspended in the original media and grown for the additional 24 h. Cell pellets were harvested by chilling samples to ∼5°C by swirling each flask in liquid nitrogen and then pelleted by centrifugation at 5,000 rpm at 4°C for 10 mins. The resulting pellets were snap frozen with liquid nitrogen.

### RNA Extraction and Quantitative Real-Time PCR

RNA was extracted from harvested samples of *F*. *diplosiphon* as previously described (Seib and Kehoe, 2002; Stowe-Evans et al., 2004) and treated with a TURBO DNA-free kit (Ambion, Austin, TX). Concentrations of isolated RNA were determined with a NanoDrop 1000 (Thermo Fisher Scientific, Waltham, MA). For each sample, 0.5 μg of RNA in 16 μl of ddH_2_O was treated with 4 μl of QuantaBio Reverse Transcriptase Master Mix (Quantabio, Beverly, MA) according to the manufacturer’s instruction. An additional aliquot of each sample was treated identically without the addition of the RT master mix for a No-RT control. After reverse transcription, each sample was diluted eight-fold. Four μl of cDNA was used for each qPCR reaction with 6 μl of Fast SYBR Green Master Mix (Applied Biosystems, Waltham, MA). qPCR was performed using a QuantStudio 7 Flex Real Time PCR System (Thermo Fisher Scientific, Waltham, MA). *orf10B*, a gene that has been shown to be consistently expressed under GL and RL (Stowe-Evans et al., 2004), was used as an internal reference gene. For a positive control in salt-stress conditions, we used *FdTSPO3* which is a full-length homolog of *TSPO* with a known role in the organism’s physiological response to salt stress (Busch and Montgomery, 2017). For the samples grown under nitrogen limitation and salt stress, the reference gene used was *rnpA*. Three technical replicates and at least four biological replicates were used for each condition. Primer sets are listed in Table 1. All qPCR procedures and analyses were performed according to the Minimum Information for Publication of Quantitative Real-Time PCR Experiments (MIQE) guidelines (Bustin et al., 2009).

**TABLE 1:** Primers for quantitative real-time PCR (qPCR).

Gene name	Forward primer/FP (5′–3′)	Reverse primer/RP (5′–3′)
*ocp1*	CGTTTACCATCGATTCAGCTCGCGG	GCTGATCCTCAGCACTCAGTTGG
*ocp2*	GATTGTGGGTAGGGAGAACATC	CTCCTTCTGCTGGTTCAGATAC
*frp*	GCATACGAGCGAGAAATCAACGCC	GCACTCAGAAAGTTATGCAACCGCC
*hcp1*	CTACAAAGAGATGGGTGGCTC	CCCTTCTGCAATATCTGAAGAAGCAG
*hcp2*	GCTCAACCCAGCGCCTCCTAACAGC	CACCACTACCACCCTTAATGAGG
*hcp3*	GCTGCTGCAGAACCCAAC	GCACGAGAATAATCGCTATCTTCGC
*ccp2*	GCGGCAACTGCTGCCTTATTTG	CGATCGCATCTGTCCCTACAAAACC
*orf10B*	AGAACTACAGCGTCAGCTTAAT	CTGCTTCGCTTTCAGCATTT

### Promoter Analysis

Using the gene search tool of the Integrated Microbial Genomes and Microbiomes (IMG/M) system available from the Department of Energy’s (DOE’s) Joint Genome Institute (JGI), the upstream intergenic DNA sequence was isolated for each *ocp* homolog to determine the region containing the gene promoter. The upstream region of *ocp2* was an exception; the intergenic region consisted of too few base pairs, requiring the sequence used to determine the promoter region to overlap with the 3′ end of the upstream gene. The SoftBerry program to predict bacterial promoters, i.e., BPROM (Solovyev and Salamov, 2011), was used to predict the operon sequence once the target sequence upstream of the ORF start was determined. The algorithm of the BPROM program predicts transcription start positions for bacterial genes regulated by sigma70 promoters. The output includes the position of predicted promoters as well as transcription factor binding sites for each predicted promoter.

## Results

### Relative Expression of *Orange Carotenoid Protein* Homologs in *Fremyella diplosiphon* Under Varied Light Conditions

**FIGURE 1:**
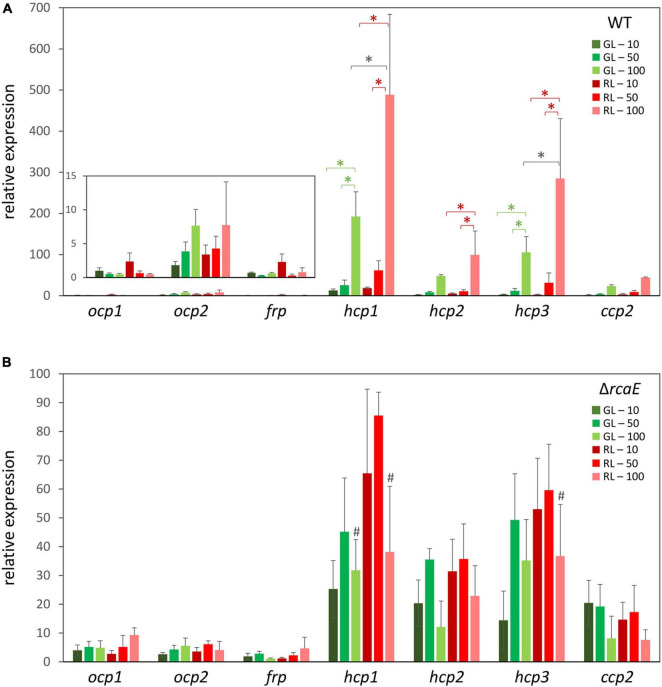
Expression of *orange carotenoid protein* (*ocp*) homologs under varying light conditions. Cultures of wild type **(A)** and Δ*rcaE* **(B)** *Fremyella diplosiphon* were grown under green light (GL) and red light (RL) at light intensities of 10 (dark green and dark red), 50 (green and red), and 100 (light green and light red) μmol m^–2^ s^–1^. Relative expression of *ocp1*, *ocp2*, *frp*, *hcp1*, *hcp2*, *hcp3*, and *ccp2* were measured using quantitative real-time PCR (qPCR). Expression under each condition is shown relative to expression under 10 μmol m^–2^ s^–1^ of green light. Error bars represent the standard deviation of each measurement. *p < 0.05, with dark gray stars indicating a difference for a strain in GL compared to RL at a specific light intensity, and red or green stars indicating a difference between different intensities of RL or GL, respectively; ^#^*p* < 0.05 representing a difference between WT and Δ*rcaE* in identical light conditions as determined by a ANOVA with Tukey-Kramer HSD *post hoc* test.

To investigate whether *ocp* homologs are controlled at the transcriptional level in response to changes in light availability, cultures of WT *F*. *diplosiphon* were grown under various intensities of green light (GL) and red light (RL) and RNA was harvested so that the mRNA levels of *ocp* homologs could be measured using qPCR. An observed tight correlation between the expression of *ocp1* and *frp* was not surprising given their adjacent positions in the *F*. *diplosiphon* genome and the fact that the FRP protein is essential for regulating the function of OCP1 (Bao et al., 2017b). Relative expression of *ocp1* and *frp* trended higher in low RL relative to low GL, and levels somewhat decreased with increased light intensity, although these differences were not significant (Figure 1A).

Distinctly, expression of *ocp2* trended upward under increased intensities of either GL or RL (Figure 1A). These differences were not significant, perhaps due to larger standard error at higher light intensities that represent conditions under which cells experience stress. Each of the remaining *ocp* homologs, *hcp1*, *hcp2*, *hcp3*, and *ccp2*, shared a common pattern of changes in expression that was consistent across several experimental conditions. For each of these genes growth under RL trended higher than GL, with a significant increase in expression for RL relative to samples grown GL at the highest light intensity specifically for *hcp1* and *hcp3* (Figure 1A). Likewise, an increase in light intensity caused an increase in mRNA levels for *hcp1* and *hcp3* in GL and all three *hcp* genes in RL. Although mRNA levels trended higher for *ccp2* at high intensity GL and RL, these differences were not significant.

### Relative Expression of *Orange Carotenoid Protein* Homologs in Complementary Chromatic Acclimation-Deficient Strains of *Fremyella diplosiphon*

Given the apparent distinctions of the impact of red versus green light on the expression levels of some *ocp* homologs, we investigated whether known CCA regulator RcaE plays a role in the photoregulation of these light-dependent genes. Prior RNA-sequencing-based analysis indicated light- and RcaE-dependent differences in mRNA counts for *ocp* homologs, at least for those homologs for which mRNA counts were detected (Table 2). Here, cultures of the CCA photoreceptor-deficient Δ*rcaE* strain of *F*. *diplosiphon* were grown under the same light conditions as the previous experiments with WT (Figure 1A) and RNA was extracted for qPCR analyses. Expression of *ocp1*, *ocp2*, and *frp* showed limited variation under various intensities of green or red light in the Δ*rcaE* strain (Figure 1B). Of note, the RL vs. GL difference in *hcp1* and *hcp3* and increased expression in response to increased light intensity observed for all three *hcp* genes in WT (Figure 1A) were no longer observed in the Δ*rcaE* mutant (Figure 1B). Specifically, the Δ*rcaE* strain exhibit lower levels of *hcp1* mRNA in both GL and RL compared to WT, and lower levels of *hcp3* mRNA in RL compared to WT for strains grown at 100 μmol m^–2^ s^–1^ (Figure 1B). Of note, samples of Δ*rcaE* grown at 100 μmol m^–2^ s^–1^ showed poor rates of growth and cultures were unable to reach the OD_750_ of 0.6 at which the other samples were harvested.

**TABLE 2:** RNA-sequencing-based analysis of expression of orange carotenoid protein (OCP), fluorescence recovery protein (FRP), helical carotenoid proteins (HCP), and C-terminal domain-like carotenoid protein (CCP) homologs in wild-type (WT) and RcaE photoreceptor-deficient strains of chromatically-acclimating *Fremyella diplosiphon* grown under green light (GL), red light (RL), or for RL vs. GL (RL:GL) counts for each strain.

	WT			Δ*rcaE*		
	GL	RL	RL:GL	GL	RL	RL:GL
*ocp1*	61	73	1.1	91	92	1
*ocp2*	ND	ND	–	147	142	0.97
*frp*	ND	ND	–	ND	ND	–
*hcp1*	164	224	1.4	150	153	1.02
*hcp2*	61	90	1.48	66	179	3.25
*hcp3*	956	1436	1.5	576	1026	1.8
*ccp2*	589	1124	1.9	1056	597	0.57

**FIGURE 2:**
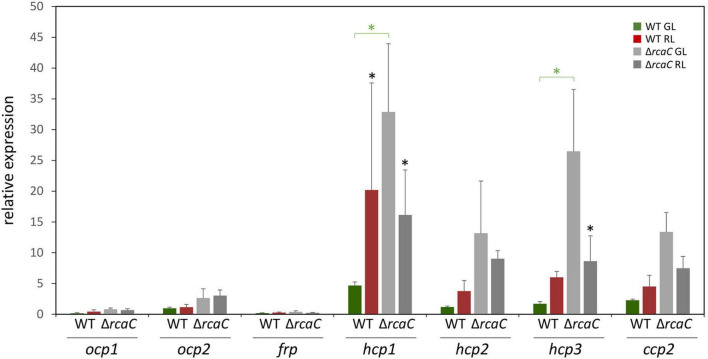
Expression of *ocp* homologs in the Δ*rcaC* strain of *F*. *diplosiphon*. Wild type and Δ*rcaC* strains of *F*. *diplosiphon* cultures were grown under green and red light at light intensities of 20 μmol m^–2^ s^–1^. Relative expression of *ocp1*, *ocp2*, *frp*, *hcp1*, *hcp2*, *hcp3*, and *ccp2* were measured using qPCR, comparing samples from the Δ*rcaC* mutant to samples from SF33. (±SD, *n* = 4). *p < 0.05, with dark gray stars indicating a difference for a strain in GL compared to RL, and green stars indicating a difference between WT and the Δ*rcaC* mutant grown in GL as determined by a ANOVA with Tukey-Kramer HSD *post hoc* test.

Two effectors, RcaF (Kehoe and Grossman, 1997), which is a small response regulator that lacks an output domain, and RcaC (Kehoe and Grossman, 1996; Li and Kehoe, 2005; Alvey et al., 2007; Li et al., 2008), which is a DNA-binding transcription factor, function downstream of RcaE in the photoregulation of pigmentation. Given the loss of light-dependent regulation of select *hcp* genes in the Δ*rcaE* strain and the central role of DNA-binding factor RcaC in direct transcriptional regulation, we also assessed transcript accumulation for *ocp* homologs in a Δ*rcaC* strain of *F. diplosiphon*. Whereas the levels of mRNA accumulation for *ocp1*, *ocp2*, and *frp* were not significantly impacted compared to WT grown in identical conditions, levels of *hcp1* and *hcp3* were significantly different in the Δ*rcaC* strain compared to WT in GL conditions (Figure 2).

### Relative Expression of *Orange Carotenoid Protein* Homologs in Low Temperature Conditions

**FIGURE 3:**
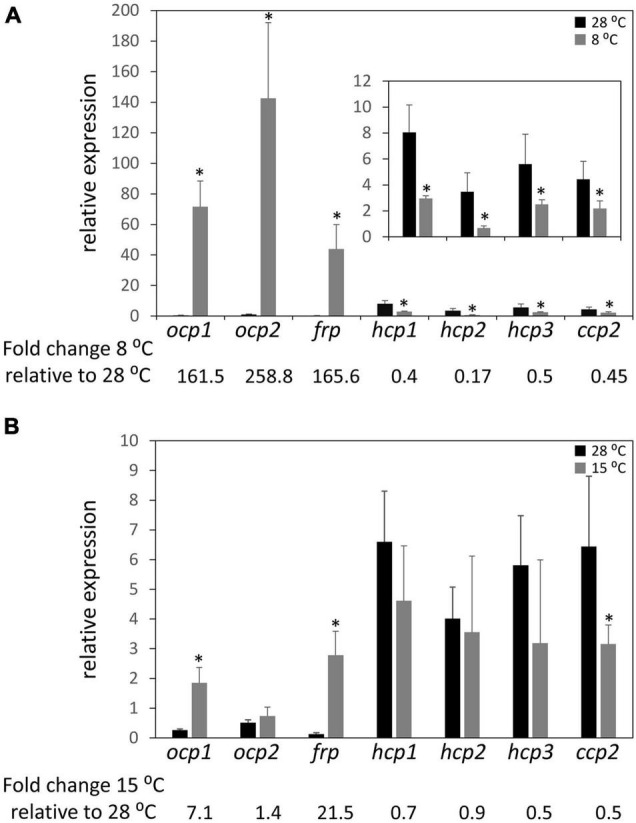
Cold-dependent expression of *ocp* homologs. Relative expression of *ocp1*, *ocp2*, *frp*, *hcp1*, *hcp2*, *hcp3*, and *ccp2* were measured using qPCR, comparing samples incubated for 24 h at **(A)** 8°C or **(B)** 15°C to control samples maintained at 28°C (±SD, *n* = 6). *p < 0.05 as determined by a two tailed Student’s *t*-test for the comparison of the treated and untreated samples.

Previous research demonstrated that the ability of OCP1 and OCP2 to reach and maintain their active (OCP^R^) state is temperature dependent (Bao et al., 2017b). Nearly 100% of OCP1 protein suspended in solution reaches the activated OCP^R^ state at both 15 and 8°C; however, the process is significantly slower for OCP1 at the lower temperature. OCP2 exhibits a distinct response to cold, with a limited portion of OCP2 (34%) reaching the activated state at 15°C, while 92% of OCP2 reaches the activated OCP^R^ state at 8°C (Bao et al., 2017b). To determine if the temperature-dependent characteristics of OCP1 and OCP2 protein are reflected in the expression of *ocp* homologs, qRT-PCR was performed with samples of *F*. *diplosiphon* grown at 28°C then transitioned to 8°C or 15°C for 24 h. At 8°C, *ocp1* and *frp* were significantly upregulated ∼160 fold compared to the control samples that were left at 28°C, while *ocp2* expression was significantly increased ∼260 fold (Figure 3A). This cold-dependent upregulation was the largest change in expression observed for any of the *ocp* family genes in this study. The three *hcp* homologs and *ccp2* were significantly downregulated ∼2 fold, or more for *hcp2*, at 8°C compared to the control samples (Figure 3A). At 15°C, *ocp1* was upregulated 7-fold and *frp* was upregulated greater than 20-fold, whereas *ocp2* was not impacted significantly (Figure 3B). None of the three *hcp* genes exhibited a significant change in mRNA levels, whereas *ccp2* was downregulated 2-fold in response to 15°C.

### Relative Expression of *Orange Carotenoid Protein* Homologs Under Salt Stress

**FIGURE 4:**
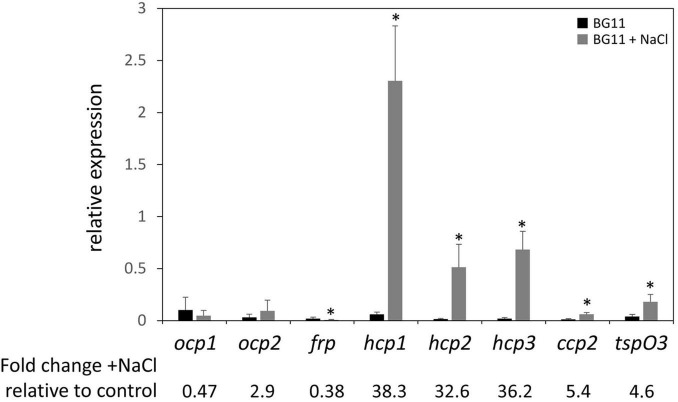
Expression of *ocp* homologs in response to salt stress. The expression levels of *ocp1*, *ocp2*, *frp*, *hcp1*, *hcp2*, *hcp3*, and *ccp2* were measured using qPCR, comparing samples incubated for 24 h in BG-11/HEPES + 0.2 M NaCl to control samples grown in BG-11/HEPES (±SD, *n* = 6). *p < 0.05 as determined by a two tailed Student’s *t*-test for the comparison of the treated and untreated samples.

Given the association of salt stress with an upregulation of *ocp* expression in *Synechocystis* sp. PCC 6803 in prior research (Fulda et al., 2006), we measured levels of mRNA for *ocp* homologs in salt-stressed *F*. *diplosiphon* cultures. With the exception of *ocp1* and *ocp2* that were not impacted and *frp* that exhibited lower mRNA accumulation in the presence of salt, expression of each *hcp* gene and *ccp2* was significantly higher in WT cultures under salt stress relative to control conditions, similar to the *F*. *diplosiphon* salt-stress marker gene *tspO3* (Figure 4). The three homologs of the N-terminal domain were the most highly over-expressed—more than 30-fold upregulation was observed for these genes.

### Relative Expression of *Orange Carotenoid Protein* Homologs Under Nitrogen-Limitation Stress

**FIGURE 5:**
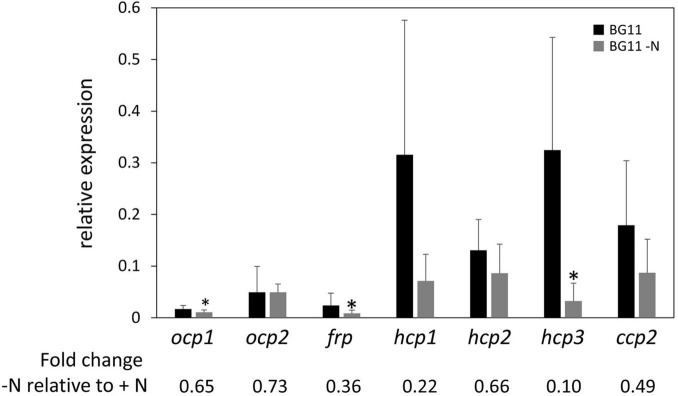
Expression of *ocp* homologs in response to nitrogen limitation stress. The expression levels of *ocp1*, *ocp2*, *frp*, *hcp1*, *hcp2*, *hcp3*, and *ccp2* were measured using qPCR, comparing samples incubated for 24 h in BG-11/HEPES without nitrogen to control samples maintained in BG-11/HEPES with nitrogen (±SD, *n* = 6). *p < 0.05 as determined by a two tailed Student’s *t*-test for the comparison of the treated and untreated samples.

Nitrogen (N) limitation has been previously associated with altered OCP protein accumulation in a proteomic study with *Microcystis aeruginosa* (Yue et al., 2015). Additionally, PBSs are degraded in response to N limitation (Collier and Grossman, 1994), suggesting that one of the roles for OCP and homologs in interacting with PBS to dissipate excess absorption of light energy may not be needed to the same degree under these conditions. Together these prior findings related to OCP and PBSs in N-limited conditions suggested to us that *ocp* homolog expression may be altered in response to N limitation. Based on qPCR analyses, expression of each *ocp* homolog trended down in WT cells exposed to N limitation compared to growth in N-replete medium. However, the difference was significant only for *ocp1*, *frp*, and *hcp3* (Figure 5).

### Relative Expression of *Orange Carotenoid Protein* Homologs in Presence of Methyl Viologen and 3-(3,4-Dichlorophenyl)-1,1-Dimethylurea

**FIGURE 6:**
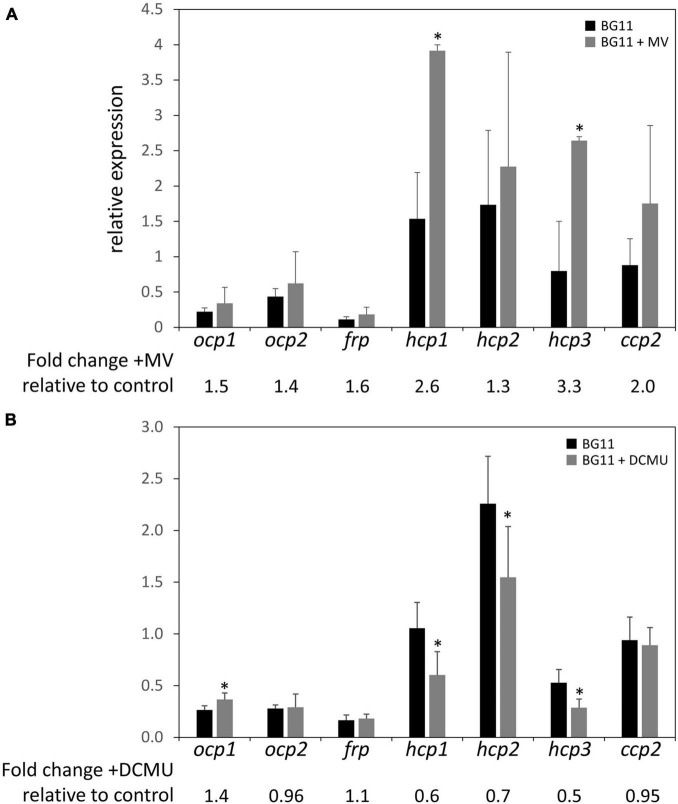
Expression of *ocp* homologs in the presence of methyl viologen or DCMU. The expression levels of *ocp1*, *ocp2*, *frp*, *hcp1*, *hcp2*, *hcp3*, and *ccp2* were measured using qPCR, comparing samples incubated in the presence of 0.03 μM methyl viologen **(A)** or 10 μM DCMU **(B)** for 24 h to untreated samples (±SD, *n* = 4–6). *p < 0.05 as determined by a two tailed Student’s *t*-test for the comparison of the treated and untreated samples.

The upregulation of some *ocp* homologs in the Δ*rcaE* strain, which exhibits high ROS accumulation relative to WT (Singh and Montgomery, 2012), as well as the general trend of increased expression of *hcp* genes in conditions that generally correspond to higher levels of cellular ROS levels led us to further examine the expression of *ocp* homologs in response to induced redox stress. To probe the impact of oxidative stress on *ocp* homologs, we treated cells with methyl viologen (MV; Fujii et al., 1990). MV acts as an artificial electron acceptor from photosystems, and thereby results in a disruption in electron transport activity that is associated with ROS generation and subsequently oxidative stress in plants (Kim and Lee, 2002) and cyanobacteria (Busch and Montgomery, 2015b; Oh and Montgomery, 2019). We conducted qPCR experiments with WT samples treated with 0.03 μM of MV and incubated for 24 h to induce oxidative stress. Expression of *ocp* homologs in the presence of MV was generally increased relative to their expression in untreated samples, although the difference observed was only significant for *hcp1* and *hcp3* (Figure 6A). The greatest increase in expression was a ∼2- to 3-fold increase observed for *hcp1* and *hcp3*.

We also simulated redox stress by treating WT cultures with 10 μM of 3-(3,4-dichlorophenyl)-1,1-dimethylurea (DCMU) and incubating for 24 h. DCMU is an herbicide that blocks the transfer of electrons from Photosystem II to the plastoquinone pool, leading to a more oxidized photosynthetic electron transport chain. The expression of *ocp1* increased significantly in the presence of DCMU compared to untreated WT samples and there was a noted decrease in the expression of *hcp1*, *hcp2*, and *hcp3* to between 50 and 70% of their expression levels in untreated samples (Figure 6B). The expression of *ocp2*, *frp*, or *ccp2* was not significantly affected by DCMU treatment (Figure 6B). A previous study described a decrease in *ocp* expression in the presence of 10 μM DCMU in *Synechocystis* sp. PCC 6803 (Maksimov et al., 2017). This shows a point of difference in the regulation of *Synechocystis ocp* and *F*. *diplosiphon ocp1*, but similarity between *hcp* homologs in *F*. *diplosiphon* with *Synechocystis ocp* in response to DCMU treatment.

### Promoter Analyses

**FIGURE 7:**
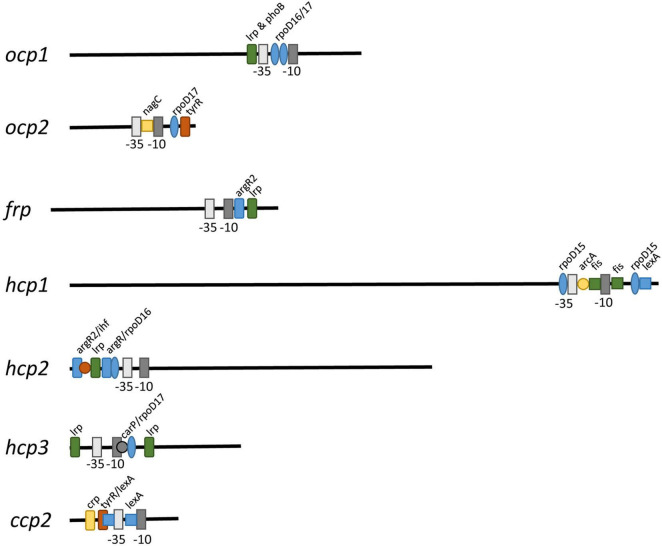
Promoter elements found in *ocp* homologs. The intergenic region upstream of each *ocp* homolog was analyzed using BPROM (Solovyev and Salamov, 2011) to predict transcription start positions (–10 and –35 sites) and transcription factor binding sites for each predicted promoter. Distinct binding sites are denoted with identical shapes and colors across distinct promoters.

To determine if some of the distinct impacts of different growth conditions on expression of *ocp* homologs were correlated with distinct transcriptional regulatory elements associated with the promoter of each gene, we conducted sequence based promoter analyses to identify conserved transcriptional elements in the gene promoters (Figure 7 and Table 3). The most common transcription factor binding sites were for *rpo*D15/16/17, with a binding site for one of these three transcription factors found in the promoter region of every gene with the exception of *frp* and *ccp2* (Figure 7). RpoD is an RNA polymerase sigma factor that functions to support initiation of transcription (Tanaka et al., 1992). Also found across multiple genes, including *ocp1*, *frp*, *hcp2*, and *hcp3*, was a *lrp* site that is frequently found to engage a leucine repressor protein that impacts cellular metabolism (Brinkman et al., 2003). Several other transcription factor binding sites were unique to the promoter region of only one or two of the *ocp* genes analyzed. An *argR2* element was found in both *hcp2* and *frp*, which is notable as this factor has been previously implicated in an unknown carbon dioxide-dependent pathway as Δ*argR2* mutants were unable to grow with CO_2_ enrichment (Nicoloff et al., 2004). Also, a *lexA* site was found in both *hcp1* and *ccp2*. The *lexA* gene encodes a transcriptional repressor involved in bacterial responses to DNA damage (Butala et al., 2009). The promoter region of *frp* had the lowest number of promoter elements.

**TABLE 3:** Promoter analyses for *ocp* homologs.

Gene	Promoter	–10 Promoter		–35 Promoter		Oligonucleotides from known TF binding sites		
	bp^a^	Sequence	bp	Sequence	bp	Name	Sequence	bp
*ocp1*	288	GTTAATACT	273	TTTACT	253	*lrp*	ATTTATTA	239
						*phoB*	TTTATTAA	240
						*rpoD16*	TTGCAAAT	264
						*rpoD17*	AATAGTTA	269
						*rpoD17*	AGTTAATA	272
*ocp2*	125	TTTTAGAAA	110	TTTCAA	91	*nagC*	ATTTTAGA	109
						*rpoD17*	TAATGTAA	129
						*tyrR*	TGTAATTT	132
*frp*	233	ATTTATTTT	218	TTAACG	198	*argR2*	TTTATTTT	219
						*lrp*	TATTTTTT	221
*hcp1*	669	TTATATATT	654	TTAACA	633	*rpoD15*	TTTTAACA	631
						*arcA*	TAACAATT	634
						*fis*	ACAATTAT	636
						*fis*	AATTATTT	638
						*fis*	TATTCTAT	659
						*rpoD15*	TAAGGTTA	673
						*lexA*	AAACCACA	680
*hcp2*	74	ACTTACTCT	59	TTTCCT	39	*argR2*	TTTTTATT	19
						*ihf*	TTTTATTT	20
						*argR2*	TTTATTTT	21
						*lrp*	TATTTTTT	23
						*argR*	TTTTTTAT	25
						*rpoD16*	TTTTTATA	26
*hcp3*	69	CTGTAAAAT	54	TGGTCA	33	*lrp*	TATTTTTT	16
						*carP*	CTGTAAAA	54
						*rpoD17*	TGTAAAAT	55
						*lrp*	TATTCTTA	81
*ccp2*	94	TTTTAGACT	79	TATATA	59	*crp*	ATCACAAA	47
						*tyrR*	AATATATA	57
						*lexA*	ATATATAC	58
						*lexA*	TTTTTTTA	76

*The BPROM program was used to identify the –10 and –35 elements, as well as conserved transcription factor (TF) binding sites with indicated conserved sequence and location in base pairs (bp) in putative promoter sequence.*

*^a^bp, base pair position with intergenic sequence assessed for conserved promoter elements.*

## Discussion

Some cyanobacteria have diversified the family of *orange carotenoid protein* (*ocp*) gene homologs. Many of the model species used for cyanobacterial species, including *Synechocystis* sp. PCC 6803 and *Synechococcus elongatus* PCC 7942, contain either one or no *ocp* genes. Thus, our understanding of the roles of multiple *ocp* homologs in a single species is limited. Initial insights into the potential biochemical activity of OCP homologs have begun to emerge. These findings include evidence that some OCP-related homologs bind PBSs to dissipate excess light energy absorption and others may function to directly quench ROS in cells (Sedoud et al., 2014; Lopez-Igual et al., 2016; Dominguez-Martin et al., 2019, 2020; Khan et al., 2020). Yet, we know very little about whether the expression of these genes are differentially controlled by distinct environmental cues that may offer insights into distinct or overlapping functions of these homologs *in vivo*.

The *ocp* gene has previously been shown to have increased expression under several stress conditions in *Synechocystis sp*. PCC 6803, including higher light intensity in studies conducted with samples grown under 20 and 300 μmol m^–2^ s^–1^ (Hihara et al., 2001). We also note differences in expression of *ocp* homologs in *F*. *diplosiphon* in response to distinct wavelengths and intensities of lights. While there are some differences relative to those reported for *Synechocystis*, the difference seen in the differential expression of *ocp1* in *F*. *diplosiphon* could be due to the lower light intensity used in our studies or associated with differences in the regulatory mechanisms controlling *ocp1* expression due to the presence of multiple *ocp* homologs in *F*. *diplosiphon*. This observation is especially true given an increase in *ocp2*, as well as *hcp1/2/3* mRNA levels in response to increased light intensity in WT.

It is notable that each of the conditions that results in increased accumulation of mRNA for the *ocp* genes (cold stress and DCMU) and the *hcp* genes (red vs. green, high light vs. low light, salt stress, and MV treatment) are also associated with higher cellular ROS levels (Singh and Montgomery, 2012). In other experiments, expression of *ocp* has been shown to increase under conditions associated with high ROS, such as high light (Hihara et al., 2001), iron starvation (Wilson et al., 2007), and the presence of another photosynthesis inhibitor DBMIB (Maksimov et al., 2017). Upregulation of *ocp* homologs under higher levels of ROS could be beneficial given that both OCP and HCP proteins have been shown to quench singlet oxygen and PBS fluorescence (Sedoud et al., 2014; Lopez-Igual et al., 2016).

The importance of regulating ROS levels in cells are critical. For example, PBS are a target of ROS in cyanobacteria (Liu et al., 2005). Thus, given the likelihood that overexcitation of PBSs or photosystems by light may result in ROS generation due to an inability to maintain photosynthetic efficiency that could subsequently result in PBS damage and impaired photosynthetic efficiency, a role for *ocp* homologs in both quenching excess light energy and ameliorating ROS accumulation is not surprising. The diversification of *ocp* homologs in some cyanobacteria is likely related to providing cellular protection related to PBS excitation and oxidative stress under dynamic conditions. OCPs and HCPs thus likely function to provide broad protection against oxidative stress and associated cellular damage across a range of stressful conditions that cells may encounter.

## Data Availability Statement

The raw data supporting the conclusions of this article will be made available by the authors, without undue reservation.

## Author Contributions

DP and PD designed and conducted the research, analyzed and interpreted the data, and contributed to writing the manuscript. BM designed the research, analyzed and interpreted the data, and wrote and edited the manuscript. All authors contributed to the article and approved the submitted version.

## Conflict of Interest

The authors declare that the research was conducted in the absence of any commercial or financial relationships that could be construed as a potential conflict of interest.

## Publisher’s Note

All claims expressed in this article are solely those of the authors and do not necessarily represent those of their affiliated organizations, or those of the publisher, the editors and the reviewers. Any product that may be evaluated in this article, or claim that may be made by its manufacturer, is not guaranteed or endorsed by the publisher.
